# Structural Features of Single-Stranded Integron Cassette *attC* Sites and Their Role in Strand Selection

**DOI:** 10.1371/journal.pgen.1000632

**Published:** 2009-09-04

**Authors:** Marie Bouvier, Magaly Ducos-Galand, Céline Loot, David Bikard, Didier Mazel

**Affiliations:** 1 Institut Pasteur, Unité Plasticité du Génome Bactérien, CNRS URA 2171, Paris, France; Stanford University, United States of America

## Abstract

We recently showed that cassette integration and deletion in integron platforms were occurring through unconventional site-specific recombination reactions involving only the bottom strand of *attC* sites. The lack of sequence conservation among *attC* sites led us to hypothesize that sequence-independent structural recognition determinants must exist within *attC* sites. The structural data obtained from a synaptic complex of the *Vibrio cholerae* integrase with the bottom strand of an *attC* site has shown the importance of extra helical bases (EHB) inside the stem-loop structure formed from the bottom strand. Here, we systematically determined the contribution of three structural elements common to all known single-stranded *attC* site recombination substrates (the EHBs, the unpaired central spacer (UCS), and the variable terminal structure (VTS)) to strand choice and recombination. Their roles have been evaluated *in vivo* in the *attI*×*attC* reaction context using the suicide conjugation assay we previously developed, but also in an *attC*×*attC* reaction using a deletion assay. Conjugation was used to deliver the *attC* sites in single-stranded form. Our results show that strand choice is primarily directed by the first EHB, but the presence of the two other EHBs also serves to increase this strand selection. We found that the structure of the central spacer is essential to achieve high level recombination of the bottom strand, suggesting a dual role for this structure in active site exclusion and for hindering the reverse reaction after the first strand exchange. Moreover, we have shown that the VTS has apparently no role in strand selectivity.

## Author Summary

Integrons play a preponderant role in the development of multiple antibiotic resistances among Gram-negative bacteria. Their success is rooted in their unique aptitude to assemble genes through a site-specific recombination process. They have recently been shown to use an unconventional recombination pathway, which involves recombination between a canonical double stranded *attI* site and a uniquely folded single stranded *attC* substrate. Due to its complex and variable structure, which includes several unpaired regions and extrahelical nucleotides, recognition of the *attC* site was elusive. Here, we determined the contribution of each of the different structural elements common to many folded single stranded *attC* substrates, in the recognition and recombination efficiency by the integron recombinase. We found that a single specific extrahelical nucleotide is responsible for the choice between the two anti-parallel complementary strands of each *attC* site, an essential discrimination for guaranteeing the expression of the acquired gene by the integron platform. These studies open the way to the design of synthetic sites and the use of this powerful loose-specificity recombination system for various gene transfer applications.

## Introduction

Integrons are DNA elements that acquire open-reading frames through site-specific recombination and convert them to functional genes by ensuring their correct expression. Integrons were first identified, in the 1980′s, as a device used by Gram negative bacteria to acquire and disseminate antibiotic resistance genes [1],[2]. More recently, the role of integrons in bacterial evolution has been extended by the identification of chromosomal integrons. Chromosomal integrons include the superintegron subfamily mainly found in *Vibrionaceae*, which can contain arrays of hundreds of various genes [3]–[6]. Unlike the integrons involved in the dissemination of drug-resistance genes, which are all associated with mobile elements, chromosomal integrons are generally sedentary components of the genome in environmental bacteria. However, the expression of both the chromosomal and mobile integron integrases is controlled by the SOS response [7] and cassette swapping between these different integrons can occur [8].

All integrons characterized to date are composed of three key elements necessary for the capture of exogenous genes: an integron integrase (IntI), a recombination site (*attI*), and a strong resident promoter (Pc) (for a review see [9],[10]). Gene cassettes correspond to a promoter-less gene associated with an *attC* recombination site [11],[12]. The *attC*×*attI* rearrangements catalyzed by IntIs lead preferentially to cassette integration at the proximal *attI* site, downstream of the resident promoter [13]. IntIs also mediate *attC*×*attC* recombination events, resulting in gene cassette deletion through circular intermediates [11].

IntI belongs to the tyrosine recombinase (Y-recombinase) family [14], which acts by forming and resolving Holliday junctions according to the “strand-swapping isomerization” model. This highly heterogeneous family catalyzes rearrangements, which are used to accomplish a variety of important biological functions [14]. The members of this family that have been studied in depth share common characteristics: the core recombination site structure and the recombination mechanism. The recombination site is invariably composed of a pair of conserved 9- to 13-bp inverted binding sites separated by a 6- to 8-bp central region and the recombination process occurs between two identical or almost identical core recombination sites (for reviews see references [15],[16]). The integron integrases form a subclass in this family, which differs from the other Y-recombinases by the presence of a specific additional protein segment required for their activity [17],[18]. Furthermore, both the *attI* and *attC* recombination sites have particular architectures which differ from the canonical Y-recombinase sites [19].

**Figure 1:**
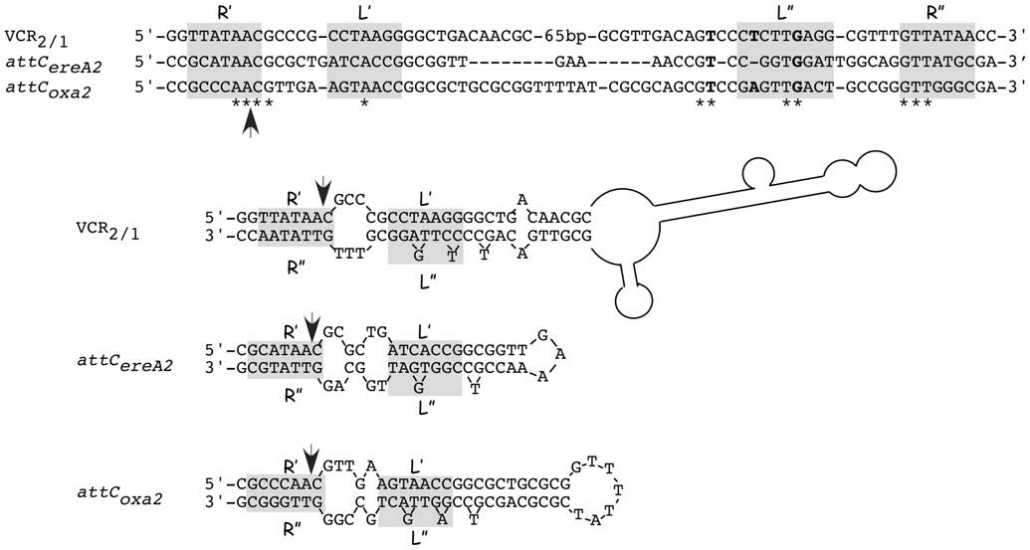
*attC* recombination sites. Sequence alignment of the bottom strands of the VCR_2/1_, *attC*_ereA2_, and *attC*_oxa2_ sites is shown. The inverted repeats L', L″, R', and R″ are indicated by grey boxes. The asterisks (*) show the conserved nucleotides between the three *attC* sites and the bold letters correspond to the three EHBs. The secondary structures of the bottom strands of the three *attC* sites are represented below and were determined using MFOLD [38]. Black arrows show the cleavage point.

The *attI* sites contain a degenerate core recombination site and, in the specific case of *attI1*, two direct repeats which seem to be required for the integrative recombination reaction [20]–[22]. The structure of the *attC* sites is more complex (Figure 1). These sites show only poor sequence conservation and their lengths vary from 57 to 142 nucleotides (nt) [23]. The sequence similarity of the *attC* sites are found at their boundaries, and correspond to two heptanucleotides boxes, now called R′ and R″ [19]. These two R boxes are part of two potential antiparallel recombination core sites, R″-L″ and L′-R′ (also termed 1L-2L and 2R-1R, respectively [23]). While recombination only occurs at L′-R′ (between the C and the adjacent A of the bottom strand R′ box), directed mutagenesis showed that R″-L″ is also essential [23]. L′ and L″ are inverted repeat sequences that show little sequence conservation with the exception of a G, specifically present in the L″ box with no complementary nt in the L′ box [23],[24]. This base is essential for the *attC*×*attC* deletion reaction to proceed [25]. The overall structure of the *attC* site is palindromic and allows the *attC* single strand (ss) to adopt a double stranded (ds) DNA-like structure by annealing of L″ to L′ and R″ to R′, which contains almost all the structural features of a canonical recombination site (Figure 1) [23],[24],[26]. For most gene cassettes, self-pairing on the same ss can be extended beyond the 7 nt R′ and R″ sequences, to a total of 9–11 consecutive complementary nt [24],[27]. It has been shown for *attC_aadA1_* [28],[29], *attC_aadA7_* and VCR_2/1_ [30], the *attC* site of the *Vibrio cholerae* superintegron cassettes, that IntI1 binds specifically to the bottom strand (bs) of a ss-*attC* DNA but not to top strand (ts) ss-*attC* DNA or to the ds-*attC* site.

We have previously shown that the ability of the IntIs to mediate recombination between the two structurally distinct *attI* and *attC* sites is driven by an unconventional recombination pathway in which the IntIs recombine their own *attI* sites under ds form, with a bs-*attC* site under ds-DNA like form [30]. The first strand exchange generates a pseudo Holliday junction, where a classical resolution by a second pair of strand exchanges would lead to the formation of covalently closed linear molecules. Productive resolution could only be achieved by replication of the pseudo Holliday junction intermediate. Indeed, structural studies have shown that the second strand exchange was prevented in the *attC*×*attC* synaptic complex and that the recombination catalytically involved only two of the four integrase monomers forming the synaptic complex [25].

**Figure 2:**
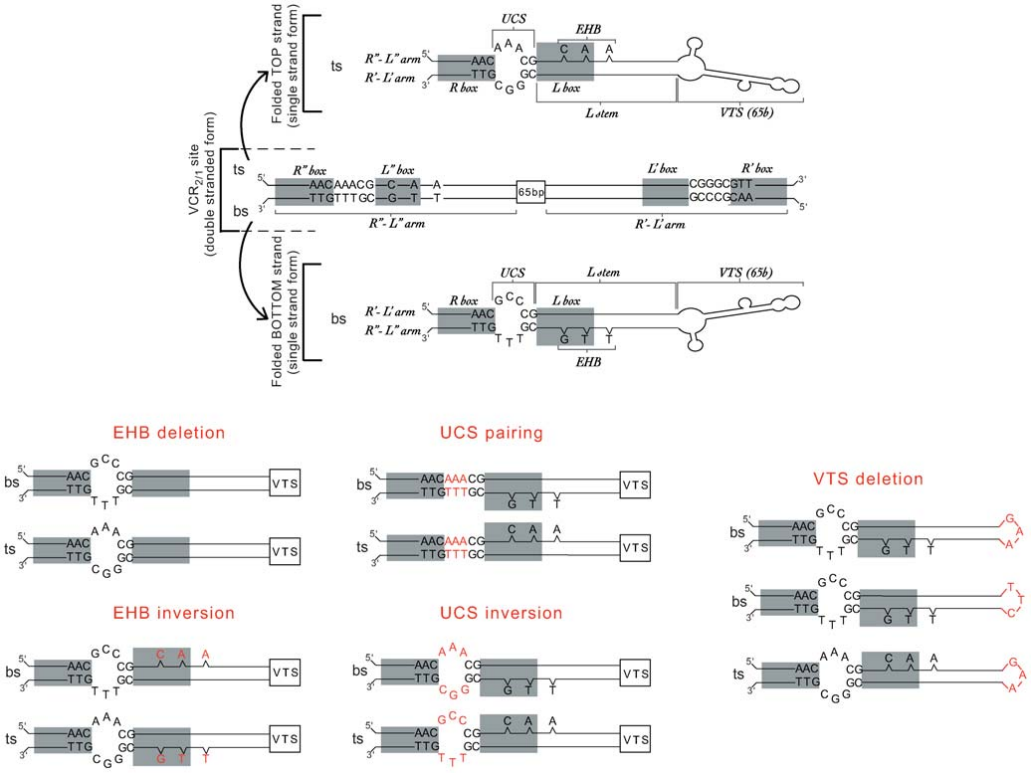
Schematic representation of the modifications introduced in the VCR_2/1_ site. The wild-type VCR_2/1_ site is shown in the double and single stranded forms. From these, the different modifications are shown: UCS inversion, UCS pairing, 65-bp VTS deletion, EHB inversion, and EHB deletion. The nt modifications are shown in red. The R″, L″, L', and R' boxes of the double strand VCR_2/1_ site and the R and L boxes of the folded single strand VCR_2/1_ site are indicated by grey boxes. UCS: unpaired central spacer, EHB: extra-helical base, VTS: variable terminal structure.

During the ds-*attI*×ss-*attC* integrative reaction strand selectivity exists, as both *in vitro* and *in vivo* data show that only the *attC*-bs could be recognized and recombined by IntI1. The *attC* conserved sequences are limited to 3 nt in both boundaries, the AAC and GTT of their R″ and R′ boxes (Figure 1). Their complementarities and their relative mirror location on each of the two strands, preclude them for ss choice determinants. The large variability shown by the rest of the sequence and the importance of the *attC*-bs folded structure led to the hypothesis that sequence independent structural recognition determinants must exist within these sites [29],[30]. The predicted *attC*-ss folded structures show that three structural features distinguish the top strand (ts) and bs-hairpins, and could therefore be responsible for *attC* strand selectivity. The annealing of the R″-L″ and L′-R′ sequences, which contain two non-complementary spacer regions, leads to the formation of the first structural element, the unpaired central spacer (UCS), which differs between both single strand hairpins (Figure 2). The second structural feature, the extra-helical bases (EHB), correspond by definition to the single bases located on the R″-L″ arm of the symmetrical *attC* sequence that have no complementary nt on the R′-L′ arm (Figure 2). Depending on the *attC* sites, there are two or three EHBs (Figure 1). Among these, the G present in the L″ box and the T found 6 to 8 nt downstream of it, are particularly conserved (Figure 1). The last structural feature is what we define as the stem terminal structure or VTS (for Variable Terminal Structure), which corresponds to the sequence located at the top of the L stem (Figure 2). The VTS varies in length among the various *attC* sites, from a predicted 3 unpaired nt to a complex branched secondary structure in the larger sites, such as the VCR site (Figure 1 and Figure S1).

**Figure 3:**
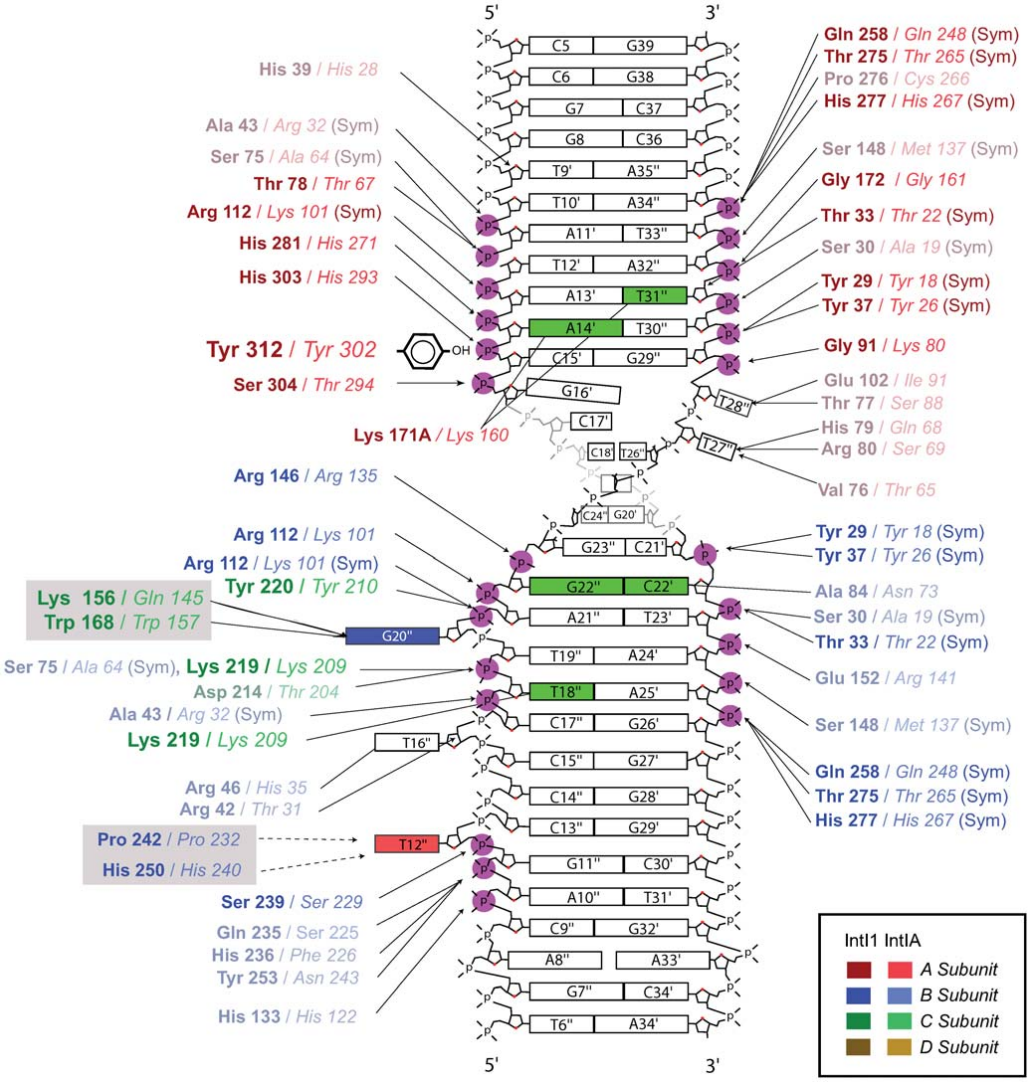
Schematic representation of the protein-DNA contacts between the IntI1 integrase and the VCRbs. IntI1 interactions with the VCRbs are derived from the model we previously made [31] based on the crystal structure of the *Vch*IntIA –VCRbs complex [25]. The IntI1 amino acids (aa) are indentified by name (three letters code) and position, while the corresponding aa in *Vch*IntIA are given in italics. The amino acids that are either not conserved between IntI1 and IntIA or among other sequenced integron integrases are faded. All hydrogen bonding protein contacts <3.5 Å are shown. Magenta circles depict protein-phosphate contacts and the position of base-specific hydrogen bonding is shown in green. The aa residues specifically involved in interaction with the EHBs G20″ (Ring stacking and hydrogen bonds) and T12″ (Stacking interactions between ring structures, dashed lines) are on grey backgrounds. The colour code for each IntI1/IntIA subunit is given in the inset. Contacts that are equivalent between the attacking and non-attacking interfaces are denoted by (Sym).

Due to their symmetrical sequence, the *attC*-ts and -bs hairpins are distinguished by these interruptions in the stem, which could direct *attC* site strand selectivity. Recently, the crystal structure of *Vch*IntIA (the *V. cholerae* superintegron integrase) bound to a substrate mimicking the folded *attC*-bs gave some indications on how integron integrases accommodate and recognize such substrates [25]. The 3D structure shows that the protein-DNA interface is almost entirely composed of non-specific protein-to-DNA phosphate interactions, and that the two EHBs (bs T12″ and G20″ in [25]), which are conserved among all the folded *attC* sites, are bound in two hydrophobic pockets and seem to be the key elements for *attC*-bs recognition and synapse assembly. Figure 3 shows the different interactions between the VCR substrate and the IntI1 residues, based on the modeling we previously made [31] from the structural study of *Vch*IntIA in complex with the same substrate [25], These two EHBs interact *in cis* and *in trans* with the recombinase monomers in order to position them along the DNA backbone and to mediate the higher order assembly of the synaptic complex.

To push these studies further and determine the single strand choice determinants involved in the integron recombination mechanism, we performed a molecular dissection of a typical *attC* site of the *V. cholerae* superintegron cassettes, the VCR_2/1_ site [3]. This site has been used in different recombination studies using both IntI1 and the *Vch*IntIA and shown to have properties similar to the other *attC* paradigm, the *attC_aadA1/7_* sites [30]–[32]. Moreover, this site was the substrate in the structural study of *Vch*IntIA [25]. Under ss form, the stem of the VCR_2/1_-hairpins contains 31-bp (R′-L′ arm) and 34-bp (R″-L″ arm) on each paired arm with an UCS composed of 5 nt from each arm (3 unpaired and 2 paired). The remainder of the folded structure, the central 65-bp, forms a large branched VTS [30] (Figure 1). Three EHBs are contained in the VCR_2/1_ ss structure (G_16_T_20_T_24_), which are conserved among the different VCR sequences and in contact with the integrase in the synaptic complex [25] (Figure 1).

Using the suicide conjugation assay we previously developed [30], we studied the role of these different structural elements of the folded ss VCR_2/1_. We present data which strongly support that the first EHB, the conserved G of the L″ box, is responsible for strand selectivity. Furthermore, we show that even if the two other EHBs are not responsible for strand selectivity, they are essential for full recombination efficiency. Finally, we assess the role of the UCS and the role of the VTS. We show that these last two structures do not participate in strand selectivity. However, we show that the orientation and the shape of the UCS are critical for the efficiency of recombination.

## Results

### Single strand choice determinants in the *attC*×*attI* recombination

Our objective was to identify the determinants of *attC* site bs-selectivity during cassette integration. The importance of the location, the orientation, and/or the sequence conservation of the different structural features of the VCR sites for strand selectivity have been analyzed by performing a series of parallel nucleotide inversions, deletions and/or substitutions. In order to facilitate the understanding of the results, the sequence modifications we tested and their classifications are shown modelled on a VCR in Figure 2. Here, “inversion” corresponds to the re-location of a single base, or of a short sequence, at the corresponding location on the mirror sequence. For example, when the EHBs are inverted, they are deleted from the R″-L″ arm and inserted in the R′-L′ arm (Figure 2). Therefore, the VCR-ts becomes more like the former VCR-bs, and vice versa. Here, “deletion” consists in the elimination of the nucleotides corresponding to the ss VCR EHBs or of the VTS (Figure 2). Finally, the “substitutions” we tested exclusively correspond to the replacement of the conserved extra-helical G_16_ by C, A or T, or to the elimination of the UCS asymmetry by making it complementary (UCS pairing, Figure 2).

**Figure 4:**
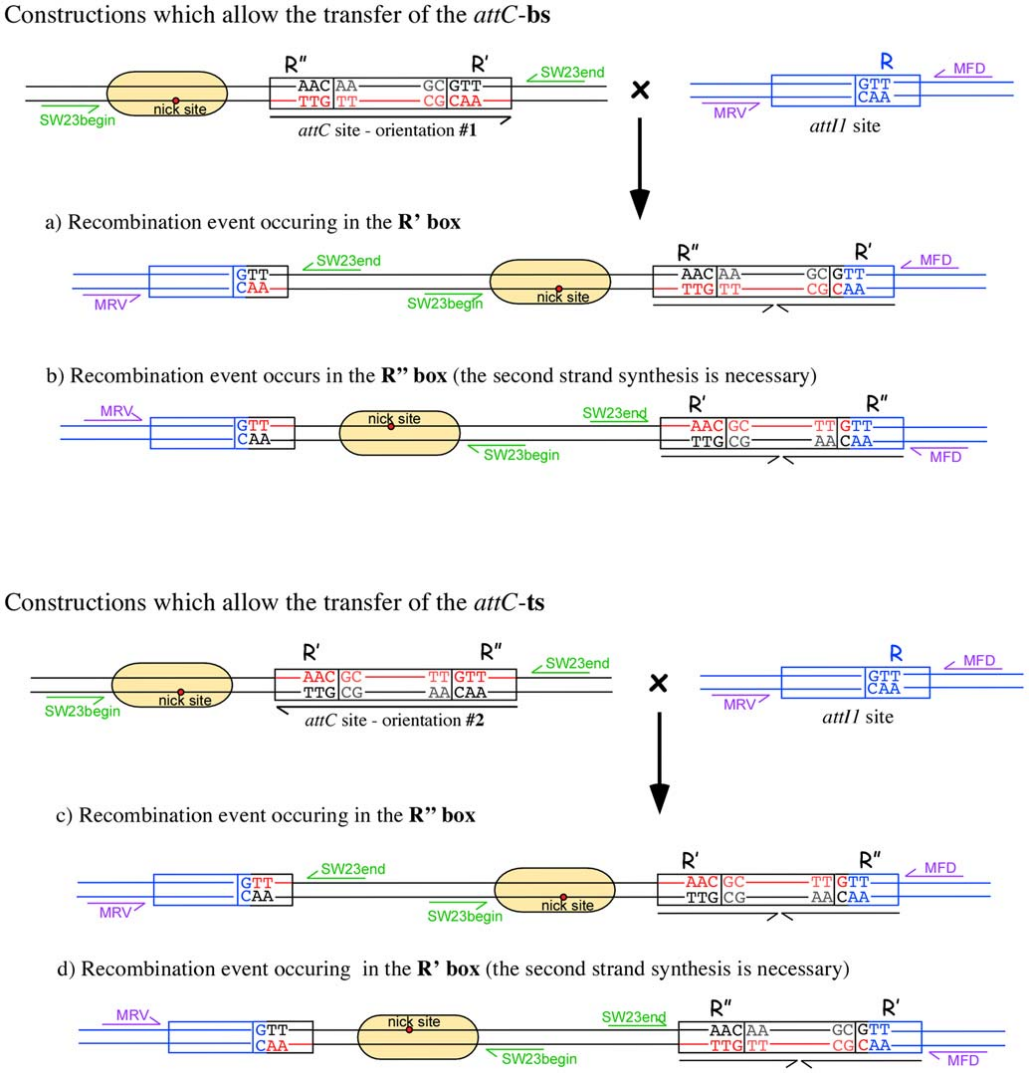
Possible recombination points of the *attC*×*attI* cointegrates and strategy to discriminate between them. The original plasmids containing the *attC* site (pSW-*attC*) and the *attI* site (pSU38-*attI1*) are represented by black and blue lines, respectively. The *attC*-bs appears in red, to facilitate the visualization of the *attC* site orientation in the cointegrates. The sequences of the recombination points of the newly formed *attC* and *attI* are shown. Four recombination events are represented: a, the recombination event occurring in the R' box after *attC*-bs transfer; b, the recombination event occurring in the R″ box after *attC*-bs transfer; c, the recombination event occurring in the R' box after *attC*-ts transfer; and d, the recombination event occurring in the R' box after *attC*-ts transfer. The positions and orientations of the oligonucleotides (SW23begin, Sw23end, MRV, and MFD) used in this study to confirm the *attC*×*attI* cointegrate formation and recombination point localization are shown in green. Note that each a, b, c, and d co-integrates has its own PCR signature.

**Figure 5:**
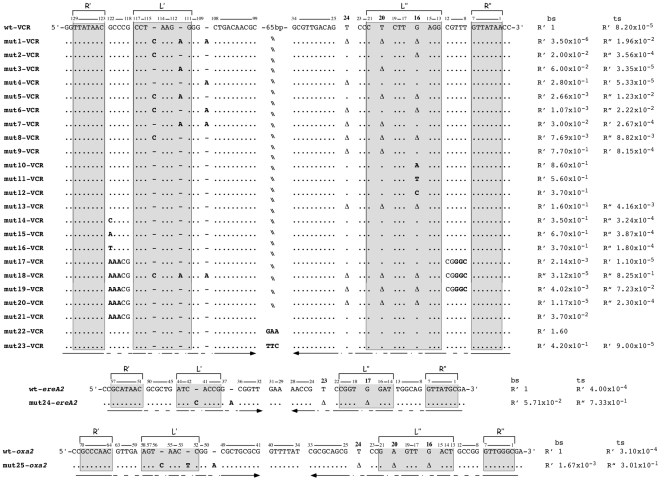
Sequence alignment of the *attC* mutant sites, and their recombination properties. The bs sequences of the wild type VCR_2/1_ (wt-VCR), *attC_ereA2_* (wt-ereA2), and *attC_oxa2_* (wt-oxa2) sites are shown, and the respective position of each nt is numbered, with position 1 as the last nt of the R″ box. Bold numbers identify the position of the EHBs. For the mutant sites, a point corresponds to an unchanged nt, Δ represents a deleted nt, and a bold letter is an exchanged nt. The R', L', L″, and R″ boxes are highlighted by grey boxes. The recombination frequencies obtained after injection of the *attC*-bs (first column) or the *attC*-ts (second column) are normalized with the wt-*attC*-bs frequency and represent a mean of three independent experiments. The location of the recombination events, R' or R″, were determined (see Materials and Methods and Figure 4). The arrows, below each scheme, show the R', L' and L″, R″ arms.

In a first set of experiments, we tested and compared the effect of the different mutations on the recombination of both the corresponding *attC*-bs and -ts using our suicide conjugation assay (see Methods as well as Figure 4 for a diagram of the possible outcomes and how one can discriminate between them by PCR). The results are represented in Figure 5. To facilitate their interpretation, the phenotypic effects of the mutations are presented as the ratio between the frequencies obtained for the tested *attC*-ts or -bs mutant and the recombination frequency of the wt-*attC*-bs. The location of the recombination point in either R′ or R″ after determination by PCR and sequencing is also given (see Materials and Methods and Figure 4). We considered that strand selectivity is shifted from bs to ts, when recombination occurred in the R″ box in all clones sequenced for each mating experiment. It is also important to stress that the *attI*×*attC* cointegrates made through recombination in the *attC* R′ box after transfer of the *attC*-ts or in the *attC* R″ box after transfer of the *attC*-bs, both correspond to recombination events involving the corresponding complementary strand, after its synthesis in the recipient strain. Furthermore, it should be noted that interestingly, for each mutated *attC* site tested, only one recombinant species was identified, corresponding to either bs or ts selectivity. Therefore, if the recombination events occur in the R′ box after the *attC*-ts injection, this would indicate that the top strand is insensitive to recombination. It also means that in this system, the injected strand is always favored even if it is less recombinogenic than the other one. Inversely, when both *attC* strands are accessible for recombination under replicative conditions, the most recombinogenic one is integrated at the *attI* site and the strand selectivity is then reduced to this strand (data not shown).

#### The wild type VCR site

In our suicide conjugation assay, VCR_2/1_ integration at the *attI1* site occurs at a rate of 1.95×10^−2^ when the bs is transferred (wt-VCR-bs) or at 1.6×10^−6^ when the ts is transferred (wt-VCR-ts) to the recipient cell, giving a bs/ts recombination rate of 1.22×10^4^. When normalized with the wt-VCR-bs frequency, the relative recombination rates are 1 for the wt-VCR-bs and 8.2×10^−5^ for the wt-VCR-ts (Figure 5). In both cases, recombination occurs in the R′ box, showing that in both cases it is the bs that is recombined, either direcly (bs injection) or after synthesis of the complementary replicated strand (ts injection) (Figure 4b and 4d).

### EHB mutations

#### EHB inversions

We inverted all three EHBs, G_16_, T_20_ and T_24_ (mut1-VCR) and observed a 2.8×10^5^-fold decrease in the recombination rate of the VCR-bs, but the recombination point remained in the R′ box, and a converse 2.40×10^2^-fold increase in VCR-ts recombination occurred, with a shift of the recombination point to the R″ box. These results show that inversion of the three EHBs at once changes strand selectivity.

To investigate the importance of each of these three EHBs, we performed sequential single base inversions. The G_16_ (mut2-VCR), T_20_ (mut3-VCR) or T_24_ (mut4-VCR) inversions lead respectively to a 50-, 16.6- or 3.6-fold decrease of the VCR_2/1_-bs recombination frequency. The mut3- and mut4-VCR-ts are recombined in the R′ box at rates similar to the wt-VCR-ts (2.4- and 1.5-fold reduction, respectively). By contrast, even though the mut2-VCR-ts is recombined only slightly more efficiently (4.3-fold augmentation) than the wt-VCR-ts, this mutation is sufficient to shift the recombination point to the R″ box. These results suggested that the extra-helical G16 alone specifies strand selectivity. To confirm these observations, we deleted the two extra helical T in mut2-VCR, (mut8-VCR in Figure 5), and found the same shift in recombination location for the ts strand. As a control we deleted these two Ts in the wt site (mut9-VCR in Figure 5) and observed that the recombination site remained in the R′ box after transfer of either of the two strands.

Starting from the G_16_ inversion (mut2-VCR), we additionally inverted either T_20_ (mut5-VCR) or T_24_ (mut6-VCR), and observed that the corresponding bs were respectively recombined 7.5- or 18.7-fold less than the mut2-VCR-bs, with the recombination point remaining in the R′ box. Conversely, we showed a 34.6- or 62.4-fold increase of the corresponding ts recombination frequency with a shift of the recombination point to the R″ box in both cases.

On the other hand, even though co- inversion of T_20_ and T_24_ (mut7-VCR) lead to a 33.3-fold decrease of the bs recombination frequency, and to a minor increase of the corresponding ts recombination (3.3-fold increase), it does not modify the location of the recombination point in the R′ box. These results demonstrate a cumulative effect of the extra-helical G and the two others EHBs (T_20_ or T_24_).

#### Extra-helical G_16_ substitution

We then substituted the extra-helical G_16_ by bases A (mut10-VCR), T (mut11-VCR) or C (mut12-VCR) to determine if the nature of the first EHB is critical in this recombination reaction. We only observed a minor decrease of the corresponding bs recombination frequency, from a 1.2- and 1.8-fold reduction for the A_16_ and T_16_ mutants, to 2.7 fold for the C_16_ mutant. Therefore, the nature of the first EHB is not essential for strand selection or the recombination efficiency.

#### EHB deletion

We then deleted the G_16_, T_20_ and T_24_ EHBs (mut13-VCR). This leads to a 6.2-fold decrease in the bs recombination frequency, but the recombination point is still located in the R′ box. Interestingly, the mut13-VCR ts is recombined at a 50.7-fold higher rate than the wt-VCR-ts and its recombination point is shifted to the R″ box. Thus the absence of EHBs does not prevent the *attC*×*attI* recombination process but leads to a partial loss of strand specificity.

### The unpaired central spacer mutations

#### UCS G_122_ substitution

The nt located just downstream of the AAC of the R′ box is a G in most *attC* sites (Figure S1). In order to test if this conservation was linked to the recombination reaction, we substituted this G (G_122_) in the VCR_2/1_ site (Figure 5), by a C (mut14-VCR), an A (mut15-VCR) or a T (mut16-VCR) and tested the properties of both strands for those three substitutions. All three have a limited impact on the recombination, with at worst a 2.8-fold reduction of the corresponding bs recombination rate and a 4.7 fold increase of ts recombination (Figure 5). Therefore, despite a strong conservation of the G_122_ in most *attC* sites, we failed to observe a significant role for this specific G in the recombination reaction that might explain this conservation.

#### UCS inversion

To probe the importance of UCS orientation in strand selectivity, we inverted the UCS (mut17-VCR). This leads to a 4.7×10^2^-fold decrease in the bs recombination frequency and to 7.5-fold decrease in the ts recombination frequency, but the recombination point remained in the R′ box for both strands. Thus, the UCS bulge orientation does not influence strand selection but its proper orientation is necessary for optimal recombination.

#### EHB and UCS co-inversion

We tested the effect of the EHB and UCS co-inversion on strand selectivity (mut18-VCR). We observed that the mut18-VCR-bs recombination rate was decreased by 3.2×10^4^-fold and that the recombination point was shifted to the R″ box, showing that recombination involved the replicated strand from the transferred bs. Conversely, we found a 1×10^4^-fold increase in the mut18-VCR-ts recombination frequency and, as previously seen after the EHBs inversion (mut1-VCR), the recombination point shifted to the R″ box. Thus the change of strand selectivity resulting from the EHBs inversion is maintained when the EHBs and the UCS are co-inverted, and ts recombination is brought to the wt-VCR-bs level.

#### EHB deletion and UCS inversion

We carried out the UCS inversion in the site lacking the EHBs (mut19-VCR) to investigate the importance of UCS orientation on the recombination rates and the location of the recombination point established for the mut13-VCR-bs and -ts. We observed the same trends as with the EHBs deletion alone, but with a much higher impact: a 2.5×10^2^-fold decrease of the bs recombination frequency, and a converse increase of the ts recombination (8.8×10^2^-fold). Interestingly, as for the EHBs deleted site (mut13-VCR), the location of the recombination point depends on the strand transferred during the conjugation event. When the bs is transferred, the R′ box is targeted, while the R″ box is targeted when the ts is transferred.

#### EHB deletion and UCS pairing

To determine if the UCS bulge shape was responsible for the variation in recombination rate observed between the -bs and -ts lacking the EHB site (mut13-VCR), we further introduced mutations in the L′-R′ spacer region (AAACG instead of GCCCG) that allow its pairing with the L″-R″ spacer in the ss form (mut20-VCR). The mut20-VCR-bs integration at the *attI1* site was drastically affected, as we observed a 8.5×10^4^-fold decrease in the recombination frequency. The mut20-VCR-ts recombination rate was slightly higher than that of the wt-VCR-ts (2.8-fold increase). As for the two previous EHB deleted VCR_2/1_ sites (mut13-VCR and mut19-VCR), the recombination point was found in the R′ box when the bs was transferred or in the R″ box when the ts was transferred. The full pairing of the UCS alone (by substitution of GCCCG by AAACG in the L′-R′ spacer region) leads to a 27-fold decrease in the recombination frequency of the bs (mut 21-VCR, Figure 5).

### Role of the VTS

To evaluate the role of the large VCR_2/1_ VTS (from nt 34 to 98, inclusive, Figure 1) on the efficiency of VCR_2/1_ integration at the *attI1* site, we replaced this 65-bp VTS by a 5′-GAA triplet (mut22-VCR) or by a 5′-TTC triplet (mut23-VCR). These substitutions only had minor effects on bs recombination: a 1.6-fold increase for mut22-VCR and a 2.4-fold decrease for mut23-VCR. When tested, the mut23-VCR-ts is recombined at a rate identical to the wt-VCR-ts site and in the R′ box as well, suggesting that the VTS does not play a role in strand selectivity for such sites.

### Study of the EHB inversion consequences in two other sites: *attC_ereA2_* and *attC_oxa2_*

The *attC_ereA2_* and the *attC_oxa2_* sites are among the smallest *attC* sites characterized to date [32],[33]. Under ss form, the predicted hairpin structures formed are almost entirely composed of a single stem (Figure 1). Their predicted VTS are reduced to an unpaired 5′-GAA triplet for the *attC_ereA2_* and to a 7 unpaired nt structure which forms a larger bulge for the *attC_oxa2_* site. They also differ by their number of predicted EHBs, 2 for *attC_ereA2_* and 3 for *attC_oxa2_*, in essentially the same position compared to VCR_2/1_. Integration at the *attI1* site occurs at rates of 1.25×10^−2^ and 2.5×10^−2^, for the *attC_ereA2_* and *attC_oxa2_* sites respectively, after transfer of the bs and of 5×10^−6^ and 7.75×10^−6^ after transfer of the ts. We co-inverted all EHBs in both sites (mut24-ereA2 and mut25-oxa2, Figure 5) and found that, as for the VCR, this leads to a polarity change; the corresponding ts becoming the preferred strand, with a recombination point located in the R″ box. These changes are accompanied by a correlated decrease in the recombination rate of the corresponding bs, the larger effect being seen with the *attC_oxa2_*-bs. However for the two mutant sites, recombination still occurs in the R′ box, as is the case for the mut1-VCR mutant (Figure 5).

### Single strand choice determinants in *attC*×*attC* recombination

**Figure 6:**
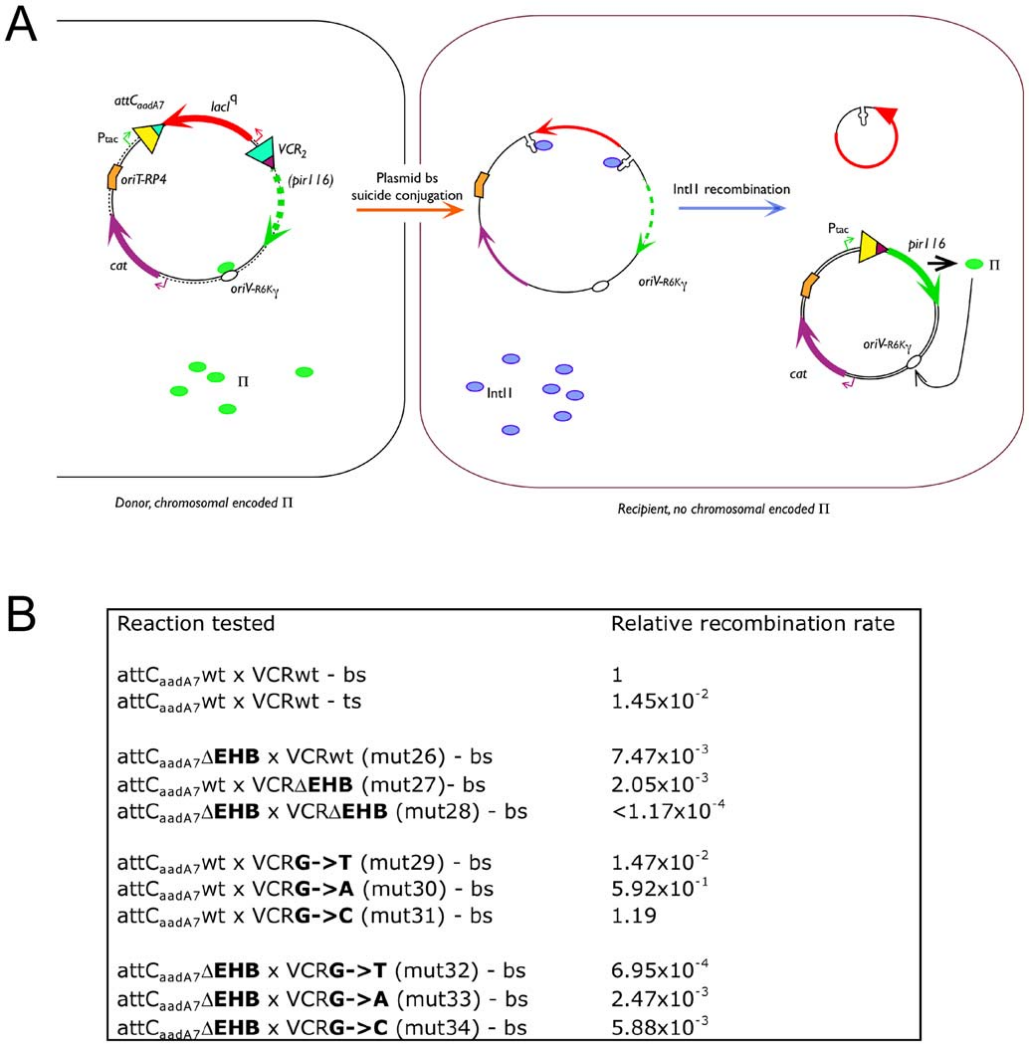
Schematic representation of the *in vivo* deletion assay and recombination frequencies of the different mutants. (A) Schematic representation of the *in vivo* deletion assay. The *in vivo* deletion assay is based on the conjugation assay previously developed for the *in vivo* integration assay. In its original form, the pSW carrying the synthetic cassette [P*_tac_*]-*attC_aadA7_*-*lacI^q^*-VCR_2/1_-pir116* is dependent on the Π protein (green ovals) produced *in trans* in the donor strain (left), as the pir116* gene is not expressed. As the recipient strain lacks the *pir* gene, the pSW plasmid replication can only be achieved after the deletion of the *lacIq* gene by an *attC_aadA7_*×VCR_2/1_ recombination reaction mediated by IntI1 (blue ovals), which allows the expression of pir116* gene from the P*_tac_* promoter. The recombination events are selected on the Cm resistant marker of the pSW plasmid. The assay is presented in more detail in the Materials and Methods. The *pir116*, *lacI^q^*, and *cat* gene are, respectively, schematized by green, red, and purple arrows, and their promoters are indicated by the same colours. The origin of replication (*oriV*_R6Kγ_) and the origin of transfer (*oriT*_RP4_) of the pSW-*attC* plasmid are shown by a white oval and an orange bent rectangle, respectively. (B) Recombination frequencies obtained after bottom (bs) or top (ts) strand injection. The recombination frequencies are normalized with attC*_aadA7_*WT×VCRWT-bs frequency and represent a mean of three independent experiments. The location of the recombination events were determined (see Materials and Methods).

In the *attC*-bs×*attI* reaction, the *attC* site is the only site that carries EHBs, since the *attI* site is only recognized in its canonical ds DNA form [28]. The 3D structure of the *attC*-bs×*attC*-bs synaptic complex with the *V. cholerae* integrase shows that the architecture of the complex could rely on the EHB binding by the integrase [25]. In order to establish if the different EHB mutations tested in the integration reaction context have similar properties in this reaction, we designed an *attC*×*attC* deletion assay, also based on conjugation as a means to deliver a suicide ss substrate. In this assay, the recombination events are selected on the establishment of a Pir^+^ phenotype that is necessary to stabilize the pSW suicide vector in the recipient strain. Expression of the *pir* gene is enabled only by an *attC*×*attC* reaction catalyzed by IntI1 that leads to the deletion of a synthetic *attC_aadA7_*-*lacI^Q^*-VCR_2_ cassette located between a P*_tac_* promoter and the *pir116* coding sequence (Figure 6A). The set-up allows transferring either of the *attC* strands depending on the relative orientation of the *oriT*_RP4_ origin of transfer (see Materials and Methods and Figure 6A). When the plasmid carries the two wt *attC* sites, cassette deletion occurs at a frequency of 3.35×10^−4^ or 4.85×10^−6^ when the transferred strand carries the *attC*-bs or -ts, respectively. In order to facilitate the understanding of the results, the frequencies measured are normalized with the attC*_aadA7_*-wt×VCR-wt frequency obtained after the transfer of their bs (Figure 6B). Therefore, the relative recombination rates are 1 for the *attC*-bs and 1.45×10^−2^ for the *attC*-ts. It should be noted that as for the integration assay, in both cases, the recombination events were localized in the *attC* R′ boxes.

We first tested the effect of the EHB deletion on *attC*×*attC* recombination since this deletion had little effect on *attC*×*attI* recombination (a ∼6 fold decrease for mut13-VCR, Figure 5). In the cassette deletion context, when made in the *attC_aadA7_* site (mut26-VCR), elimination of the EHBs leads to a reduction of 1.3×10^2^-fold in the deletion frequency or to a 4.9×10^2^-fold reduction when the EHBs were deleted from the VCR_2/1_ site (mut27-VCR, Figure 6B). Furthermore, when performed in both sites at once (mut28-VCR), deletion of the EHBs completely abolishes cassette deletion (Figure 6B).

Since the extra-helical G_16_ substitution by T, A or C had a minor effect on the integration of the VCR_2/1_ site at the *attI1* site (a 37% reduction for the G>C substitution in mut12-VCR, Figure 5), but seemed to play a key role in the establishment of the *attC*-bs×*attC*-bs synaptic complex, we tested the effect of these substitutions on cassette deletion efficiency. We introduced them in the VCR site, as in the integration assay, while the *attC_aadA7_* was either left unmodified (mut29-VCR, mut30-VCR and mut31-VCR) or deleted of its two EHBs (mut32-VCR, mut33-VCR and mut34-VCR). In conjunction with the wt *attC_aadA7_* site, the different substitutions have very different effects (Figure 6B). While, the G>C substitution (mut31-VCR) seems to have a limited impact on the deletion frequency (1.2-fold increase), the G>A substitution (mut30-VCR) leads to a minor decrease of the deletion frequency (1.7-fold). On the other hand, the G>T substitution (mut20-VCR) leads to a sizable decrease of the deletion frequency (68-fold). Finally, when coupled to *attC_aadA7_* ΔEHB, both effects are superimposed. The G>T substitution (mut32-VCR) had a larger effect, with a relative frequency decrease to 6.95×10^−4^ (10.7-fold in comparison to *attC_aadA7_*ΔEHB×VCRwt). The G>A substitution (mut33-VCR) decreased the *attC_aadA7_*ΔEHB effect by a factor of ∼3 and the G>C (mut34-VCR) has only a minor effect (1.3-fold decrease) (Figure 6B). The effects of EHBs deletion and/or EHB substitution are thus amplified in the *attC*×*attC* recombination when compared to those in the *attC*×*attI* recombination context.

## Discussion

### Directionality of the recombination process

In site-specific recombination systems, the polarity of the partner sites and their relative orientations are essential for the biological functions embedded in the recombined DNA. Indeed, it precisely directs the choice between deletion and inversion of DNA segments in intramolecular recombination reactions and determines the orientation of the integrated DNA sequences in intermolecular recombination events.

For most members of the Y-recombinases family which have been studied in detail, it is the spacer present between the two recombinase-binding elements of the core site that establishes the overall polarity of the sites [34]. Sequence homology in the spacer of the two sites is absolutely required and its asymmetry dictates the directionality of the recombination event. The creation of heterologies between the two partner spacers abolishes the isomerization step and the second strand exchange cannot occur. The simplest systems, exemplified by the Cre and Flp recombinases, do not require accessory factors. Indeed, the core recombination site contains all the information necessary to complete the recombination reaction. In more complex systems, the polarity is also encoded in the recombination locus, but it also involves accessory sequences and proteins that allow the assembly of a defined complex and drive the overall reaction in a given direction.

Such a polarity in the recombination process also governs the integron system. The cassettes are usually integrated in only one of the two possible orientations within an integron, the one allowing expression of the carried genes from the Pc promoter. For the integron, the recent discovery that cassette recombination involves a single strand substrate has raised a number of questions regarding the specific recognition of the *attC* bottom strand as the substrate for recombination. Indeed, the polarization of the integron recombination system is based on strand selectivity, the bs being recombined rather than the ts. How is this selectivity brought about? Since no accessory factor seems to be required for the recognition and recombination of the *attC* site [25], the polarity had to be encoded in the *attC* core recombination site. The lack of sequence identity among the various *attC* sites (Figure S1) and the overall conservation of the *attC*-bs folded structure, independent of its sequence, led to the hypothesis that sequence-independent structural recognition determinants must exist within *attC* sites [29],[30]. This assumption was strengthened by the structural studies which showed that the protein-DNA interface was almost entirely composed of non-specific protein-to-DNA phosphate interactions (Figure 3) [25],[31].

Due to the intrinsic symmetry of the *attC* sequence, the predicted *attC*-bs and -ts secondary structures only differ by three conserved structural features, the unpaired central spacer (UCS) separating the R and L boxes, the two to three extra-helical bases of the L stem (EHB) and the variable terminal structure located at the top of the L stem (VTS). Using *in vitro* binding experiments between derivatives of the *attC_aadA1_*-ts or -bs and IntI1, Sundström and colleagues identified several alterations resulting in ts recognition with a reciprocal loss of binding to the bs [29]. They mainly found that transfer of the EHB from the R″-L″ arm to the R′-L′ arm and co-inversion of the VTS 5′-GAA triplet were necessary to obtain full binding to the *attC_aadA1_*-ts oligonucleotide. We present here an *in vivo* study of the role played by the three conserved structural features, using our suicide-conjugation assay [30] to deliver the modified *attC*-ts or -bs into a recipient cell expressing the IntI1 integrase and carrying the *attI1* partner site [30]. This assay allows us to study the recombination reactions in biological conditions and in the presence of the partner *attI* site. In contrast, the oligonucleotide binding-based experiments only concern a single conformational state of the site that, although useful, is far from its biological state. We studied the VCR site, one of the *attC* paradigms and for which we know the 3D structure of the *attC*-bs×*attC*-bs synaptic complex.

### The distinct roles of the *attC* sites′ structural features

#### The extra-helical G_16_ specifies the strand selectivity

After conjugation, the relative recombination rate of after bs and ts injection differed by 1.22×10^4^-fold. In both cases the recombination point was located in the R′ box, showing that after ts transfer, recombination occurred from the replicated complementary bs.

We found that inversion of the G_16_ alone (mut2-VCR), is sufficient to lead to the relocation of the recombination point from the R′ to the R″ box when the ts is transferred showing that it now the injected ts that is directly recombined, while, neither the inversion of T_20_ or T_24_ alone (mut3-VCR and mut4-VCR), nor the T_20_ T_24_ co-inversion (mut7-VCR), change the R′ box recombination point location after ts injection (Figure 5). These results demonstrate that G_16_ is responsible for strand selectivity. However, even if they do not intervene at this step, inversion of T_20_ T_24_ increases the recombination frequency of the ts with a reciprocal decrease of the bs recombination efficiency. The results obtained with the *attC_ereA2_ and attC_oxa2_* sites, when all the EHBs were co-inverted (mut24-ereA2 and mut25-oxa2) show that this rule can likely be extended to other *attC* sites.

One should notice however, that the recombination rate of the inverted EHB mutant ts remains lower than the corresponding WT *attC*-bs, (for instance, at best 50-fold lower than WT-bs, for the VCR_2/1_ derivatives). In addition, when the *attC*-bs of the different G_16_ inverted mutants are delivered by conjugation, one observes that the recombination event, though in most cases occurring at a frequency lower than the corresponding ts, always involves the R′ box. This observation suggests that the rest of the structure, most certainly the UCS (see below) keep an imprint for the strand recognition.

#### The nature of the first EHB is not essential for the VCR_2/1_ recombination

The alignment of hundreds of *attC* shows that the first EHB is almost exclusively a G ([24] and Figure S1). This conservation together with the specific interactions made with the *Vch*IntIA integrase in the *attC*×*attC* synaptic complex [25] led us to test if its substitution by a different base would be deleterious to the recombination efficiency in our assay. We therefore substituted G_16_ by an A, a T or a C (Figure 5). We only observed at most a 2-fold decrease of the bs recombination efficiency. When tested in the context of our *attC*×*attC* deletion assay, we also observed a limited effect, though different from those obtained in the *attI*×*attC* reaction. Indeed, the only notable effect was seen for the G>T substitution which reduced the deletion rate to ca 1.5% of WT. We also tested these 3 substitutions with the partner *attC_aadA7_* site devoid of its EHB. Deletion of the EHB in the *attC_aadA7_* decreased the deletion frequency to less than 1% of the WT frequency, and we found that the G_16_ substitution in the second site had the same effect to that observed when coupled to the WT *aadC_aadA7_* (Figure 6B).

#### Deletion of the EHB leads to a partial depolarization of the VCR_2/1_ site

Deletion of the three VCR EHBs leads to a depolarization of the site, visible in our suicide recombination assay (mut13-VCR, Figure 5). However, this depolarization is only partial as the mut13-VCR-ts recombination frequency is ∼40-fold lower than the mut13-VCR-bs.

Interestingly, we had previously shown that the *attC*×*attC* deletion recombination reaction catalyzed by *Vch*IntIA cannot occur in the absence of the EHBs and in particular not in the absence of the conserved G_16_ [25]. We found the same inability for IntI1 to perform the *attC*×*attC* recombination when both *attC* sites are devoid of their EHBs (Figure 6), while deletion of the EHBs in only one of the two sites decreased the relative recombination frequency to, at most, 7.10^-3^ of the WT one (Figure 6B). Recognition and binding of the *attI* site by the integrase follow different rules. Even if one ds site lacks EHBs, deletion of the EHBs in the partner *attC* sites affected the integration reaction to a lesser extent (∼6 fold decrease) than the *attC*×*attC* intra-molecular recombination reaction, where deletion of the EHBs completely prevents recombination. Interestingly, in previous work we found that annealing of the EHBs also decreases the bs integration frequency to a greater extent than EHBs deletion (VCR_GTT_ mutant in [31]). We also isolated an IntI1 mutant, D161G, that has a higher integration activity at the *attI* site with both the EHBs annealed and the EHBs deleted sites. This mutation is located in a loop which is very important for inter-subunit protein/protein interactions and our data suggested that the D161G mutation, which should allow more flexibility for synaptic complex assembly, compensates for the rigidity brought by the full annealing of the L′/L″ box in these sites [31].

#### The UCS bulge orientation and its shape play an important role for the recombination efficiency

Not surprisingly, the spacer regions of the partner sites, which are not conserved among the various *attC* sites (apart for the G located just downstream of the AAC of the R′ box, G_122_ in Figure 5) or the different *attI* sites, do not intervene in the establishment of strand selectivity. Substitution of the conserved G_122_ had little effect when tested in the *attI*×*attC* reaction, and the reason for its conservation remains obscure. However, the orientation and bulge shape of the UCS influence the recombination efficiency. Indeed, we noticed a significant decrease in VCR_2/1_ integration at the *attI1* site when we inverted the UCS (mut17-VCR) and when we rendered it fully annealed in the ss structure (mut21-VCR), respectively. In our previous study [31] we selected an IntI1 mutant, P109L, which recombined the UCS-inverted VCR site with only a 10-fold and 5-fold decrease compared to the WT site and the fully annealed UCS VCR site, respectively. Again, this mutation, located in the linker between the N-terminal domain and the catalytic domain, is predicted to give more flexibility to the synaptic complex assembly.

The UCS bulge is essential for the recombination reaction in the absence of the EHBs. Indeed, with our suicide conjugation assay, when all EHBs were deleted and the UCS was mutated to make it fully paired (mut20-VCR), the ds-*attI1*×ss-*attC* recombination process was drastically affected for both strands, but the ΔEHB depolarization effect remained (Figure 5). In the same ΔEHB context, inversion of the UCS (mut19-VCR) led to an inversion of polarity in the recombination site. These results suggest that in absence of the extra-helical G_16_, T_20_ and T_24_, the UCS of the VCR_2/1_ site can still impose an *attC* strand selectivity in the *attI1*×*attC* recombination reaction.

#### The VCR_2/1_ large VTS is not involved in strand selectivity

The VTS is the most variable part among the different *attC* sites. We did not observe any effect on the location of the recombination point and very little variation in the recombination frequency when the VTS was deleted (mut22-VCR and mut23-VCR, Figure 5). Sundström and colleagues had observed that a GAA substitution by TTC in the *attC_aadA1_*-bs VTS severely decreased the IntI1 binding *in vitro* [29]. Our *in vivo* results on the VCR site are clearly different and suggest that this discrepancy may reflect a feature specific of *attC_aadA1_*.

### Control of the recombination

Specific recognition of the *attC* bottom strand is the first challenge for the *attI-*ds×*attC*-bs and *attC*-bs×*attC*-bs recombination processes. The second issue in these processes is targeting the R′ box for the strand exchange. In the integron reaction the recombination catalytically involves only two of the four integrase monomers forming the synaptic complex, as there is no second strand exchange. The crystallographic structure of the *V. cholerae* integrase *attC*-bs synaptic complex reveals the *in cis* IntI binding to the T_24_ EHB, and the consequent disorganization of the catalytic site, as the main factor for halting the reaction after the first strand exchange [25]. In the *attI*-ds×*attC*-bs reaction, the first strand exchange must occur in the R boxes of both *attC*-bs and *attI*-bs to maintain the integrity of both sites after the recombination event. How is the recombination point targeted? The *attI* site has no characteristic EHB to specifically differentiate its R box from its L box. Moreover, no accessory factors seem to be required for the recognition and the recombination of the *attC* site. Therefore, an active site exclusion is required.

This polarity is likely driven by several factors. The first is likely the fact that the L box found in different characterized *attI* sites is never identical to the GTT (ts)/AAC (bs) R box consensus. In particular, the G/C is never found, preventing the exchange. The second factor is presumably the extra-helical T_24_ binding which disorganizes the catalytic site by pulling the catalytic tyrosine farther from the phosphate chain in the two IntI monomers bound to this EHB *in cis* [25]. However, its presence is neither essential for the *attC*×*attC* deletion reaction, with only a 10- fold decrease [25], nor for the *attI*×*attC* integrative process, as we found it to occur at the right place with a wt recombination efficiency (data not shown).

Here, we provide evidence suggesting that a third factor, the *attC*-bs UCS, could be partly responsible for the active site exclusion by directing the initial bending of the site. It is known that site bending is essential to control the outcome of recombination in canonical site-specific recombination model [16]. Here we found that UCS and EHBs co-inversion are necessary to obtain full exchange of strand selectivity (mut17-VCR and mut18-VCR). Furthermore, when the *attC* site is devoid of its EHBs, our results show that it is sufficient to maintain a bs polarity over the ts.

The UCS bulge shape is thought to increase the flexibility of the core recombination site [25], and its asymmetric nucleotidic composition – A rich in the R″-L″ arm and GC rich in the R′-L′ arm – seem to influence the initial bending orientation of the VCR_2/1_ site. We previously selected an IntI1 mutant (P109L, [31]) that recombined the UCS inversion mutant (mut13-VCR) at a rate almost equivalent to that of IntI1 on the wt VCR. This mutation is located in the non-structured region linking the N-terminal domain to the catalytic C-terminal domain, in the C-shaped 3D structure. Several properties of the P109L mutant suggest that this mutation affects the flexibility of the integrase and allows for compensation of the structural change of the UCS bulge of the mutant site [31].

The UCS bulge shape could also prevent strand exchange reversal. An earlier study of the Flp-*frt* system showed that the presence of mismatches adjacent to the recombination point can favor cleavage over religation of the substrate [34]. This property can be essential for the success of the first strand exchange in the unusual integron single-stranded recombination process where this exchange is carried out similarly to those of other Y-recombinases but resulting in a pseudo Holliday junction. Holliday junction formation in absence of homology in the spacer has previously been demonstrated using a synthetic λ *att*-site [35]. Nevertheless, the heterologies present in the spacer segments seem to interfere with the isomerization step, and therefore a rapid reversal of the strand exchange is observed. The existence of a UCS could restrain the return to the initial substrate before the resolution of the pseudo Holliday junction by the host. The decrease in the bs recombination frequency in the VCR_2/1_ UCS pairing (mut21-VCR) could thus at least partly reflect the importance of this reverse effect. However, it is difficult to separate this from the probable effect of UCS annealing on the bending of the site. More work will be necessary to clarify this point.

In conclusion, our study shows that two of the three structural features (EHB and UCS) shared by all *attC* sites play key roles for the integron recombination reaction. The first and absolutely conserved EHB, G_16_ in VCR_2/1_, is essential for strand selectivity and therefore for the expression of the genes carried in the cassettes. The unpairing of the UCS and its asymmetry are essential for proper synaptic assembly, and certainly for ensuring the success of the consecutive steps of the single strand recombination process. We did not see any significant effect of the large VCR VTS in our conjugative assay, where the *attC* site is delivered as single strand substrate for recombination. But, we cannot exclude the possibility that the VTS play a major role in the *attC* folding in replicative conditions, and this is currently under investigation. On the other hand, more extensive work will be performed on other structurally different *attC* sites to determine if the functions assigned to the different structural features of the VCR sites, can be extended to others *attC* sites.

Finally, these results establish that the folded single strand structure of the *attC* site is important for integron recombination and confirms the originality of this mechanism for gene capture and dispersal.

## Materials and Methods

### Bacterial strains, plasmids, and media

Bacterial strains used in this study are DH5α (Laboratory collection), Π1, β2163 [36], UB5201 [37] and UB5201-Pi [30]. Plasmids are described in Table 1. *Escherichia coli* strains were grown in Luria-Bertani (LB) or, when specified, in Muller-Hinton (MH) broth at 37°C. Antibiotics were used at the following concentrations: ampicillin (Ap), 100 µg/ml; chloramphenicol (Cm), 25 µg/ml; kanamycin (Km), 25 µg/ml; nalidixic acid (Nal), 30 µg/ml. Thymidine (Thy) and diaminopimelic acid (DAP) were supplemented when necessary to a final concentration of 0.3 mM. Isopropyl-β-d-thiogalactopyranoside (IPTG) was added at 0.8 mM final concentration. Chemicals were from Sigma.

**Table 1:** Plasmids used in this study.

Plasmid number	Plasmid description	Relevant properties and construction
p929	pSU38Δ::attI1	*orip15A* [Km^R^], [32]
p1394	pTRC99A::*intI1*	*oriColE1*, [Ap^R^], [4]
p970	pSW23T	*oriV*_R6Kγ_, *oriT*_RP4_;[Cm^R^], [36]
p2637	pSW23T_ISS_	pSW23T::*oriT*_RP4_ with *Eco*RI and *Bam*HI restriction site inversed; *oriV*_R6Kγ_ [Cm^R^] [30]
p1880	pSW23T::VCR_2/1_ (BOT^i^)	[32]
p2656	pSW23T::VCR_2/1_ (TOP^ii^)	[32]
p3615	pSW23T::attC_ereA2_ (BOT^i^)	Annealing of wt-ereA2-rev and -fwd and *Eco*RI – *Bam*HI cloning into pSW23T
p4392	pSW23T::attC_ereA2_ (TOP^ii^)	Annealing of wt-ereA2-rev and -fwd and *Eco*RI – *Bam*HI cloning into pSW23T_ISS_
p3616	pSW23T::attC_oxa2_ (BOT^i^)	Annealing of wt-oxa2-rev and -fwd and *Eco*RI – *Bam*HI cloning into pSW23T
p4390	pSW23T::attC_oxa2_ (TOP^ii^)	Annealing of wt-oxa2-rev and -fwd and *Eco*RI – *Bam*HI cloning into pSW23T_ISS_
P1177	pSB118::pir116*	[36]
p4699	pTRC::pir116*	*Eco*RI/*Bam*HI fragment amplified in a sequential manner [3-steps] from p1177 with *pfu* DNA polymerase and with the pirA1/pirA2, pirB1/pirB2, pirC1/pirC2 couple of primers in pTRC99A
p1187	pSU38::*attC_aadA7_*-*catT4*-VCR2	[31]
p4700	pSU38::*attC_aadA7_*-VCR_2_	Deletion of the catT4 gene of the p1187 by inverse PCR with 1187-ΔCat-1 and 1187-ΔCat-2 primers. Circularisation of the PCR product by *Eco*RV digestion, following by ligation.
p4701	pSU38::*attC_aadA7_*-VCR_2_-pir116*	*Sph*I/*Hind*III PCR fragment from p4699 in the p4700 digested by *Sph*I/*Hind*III.
p4702	pSU38::*attC_aadA7_*-*lacI^q^*-VCR_2_-*pir116*	*Kpn*I/*Xba*I fragment amplified from pTRC99A with the lacI^q^-1 and lacI^q^-2 primers in p4700.
p4703	pSW23T::[P*_tac_*]-1	*Mfe*I/*Bam*HI PCR fragment amplified from pTX1k [39] with pTAC(pTX1)-1 and pTAC(pTX1)-2 primers in pSW23T.
p4704	pSW23T::[P*_tac_*]-2	*Mfe*I/*Bam*HI PCR fragment amplified from pTX1k [39] with pTAC(pTX1)-1 and pTAC(pTX1)-2 primers in pSW23T_ISS_
p4746	pSW23TΔBamHI::[P*_tac_*]-1	*Sac*I fragment from the p4703 religated on itself
p4747	pSW23TΔBamHI::[P*_tac_*]-2	*Sac*I fragment from the p4704 religated on itself
p4781	pBBR-MCS4::*attC_aadA7_*-*lacI^q^*-VCR_2_-pir116*	*Eco*RI/*Hind*III fragment from p4702 in pBBR-MCS4 digested by *Eco*RI/*Hind*III
p6944	pSW23T::[P*_tac_*]-*attC_aadA7_*-*lacI^q^*-VCR_2_-pir116* (BOT^i^)	*Eco*RI/*Sal*I fragment from p4781 in p4747 digested by *Eco*RI/*Sal*I
p4742	pSW23T::[P*_tac_*]-*attC_aadA7_*-*lacI^q^*-VCR_2_-pir116* (TOP^ii^)	*Eco*RI/*Sal*I fragment from p4781 in p4746 digested by *Eco*RI/*Sal*I
p6945	pSW23T::[P*_tac_*]-*attC_aadA7_*-*lacI^q^*-VCR_2_ **Δ_EHB_**-pir116* (mut27)	Mutagenesis by PCR on the p6944 with the VCR(ΔEHB) and SDM-VCR-ΔEHB primers
p6946	pSW23T::[P*_tac_*]-*attC_aadA7_* **Δ_EHB_**-*lacI^q^*-VCR_2_-pir116* (mut26)	EcoRI/BamHI fragment obtained by annealing between the Ad7-ΔEHB-1 and Ad7-ΔEHB-2 primers filling with the Taq polymerase, digestion and cloning in p6944
p6947	pSW23T::[P*_tac_*]-*attC_aadA7_* **Δ_EHB_**-*lacI^q^*-VCR_2_ **Δ_EHB_**-pir116* (mut28)	EcoRI/BamHI fragment from p6946 in p6945 digested by *Eco*RI/*Bam*HI
p6948	pSW23T::[P*_tac_*]-*attC_aadA7_*-*lacI^q^*-VCR_2_ **[G_16_→C]**-pir116* (mut31)	Site-directed mutagenesis by PCR on the p6944 with the VCR(G_16_→C) and SDM-VCR primers
p6949	pSW23T::[P*_tac_*]-*attC_aadA7_* **Δ_EHB_**-*lacI^q^*-VCR_2_ **[G_16_→C]**-pir116* (mut34)	EcoRI/BamHI fragment from p6946 in p6948 digested by *Eco*RI/*Bam*HI
p6950	pSW23T::[P*_tac_*]-*attC_aadA7_*-*lacI^q^*-VCR_2_ **[G_16_→T]**-pir116* (mut29)	Site-directed mutagenesis by PCR on the p6944 with the VCR(G_16_→T) and SDM-VCR primers
p6951	pSW23T::[P*_tac_*]-*attC_aadA7_* **Δ_EHB_**-*lacI^q^*-VCR_2_ **[G_16_→T]**-pir116* (mut32)	EcoRI/BamHI fragment from p6946 in p6950 digested by *Eco*RI/*Bam*HI
p6952	pSW23T::[P*_tac_*]-*attC_aadA7_*-*lacI^q^*-VCR_2_ **[G_16_→A]**-pir116* (mut30)	Site-directed mutagenesis by PCR on the p6944 with the VCR(G_16_→A) and SDM-VCR primers
p6953	pSW23T::[P*_tac_*]-*attC_aadA7_* **Δ_EHB_**-*lacI^q^*-VCR_2_ **[G_16_→A]**-pir116* (mut33)	EcoRI/BamHI fragment from p6946 in p6952 digested by *Eco*RI/*Bam*HI

i BOT: Bottom strand of the *attC* site transferred by conjugation.

ii TOP: Top strand of the *attC* site transferred by conjugation.

### pSW-*attC* construction procedure

VCR mutant sites were constructed by annealing of two complementary partially overlapping primers followed by a filling in step using the Taq DNA polymerase (Promega) according to the manufacturer′s instructions. Primers were obtained from Sigma-Proligo (France) and are listed in the Table S1. The conditions used for the annealing - filling procedure were as follows: 94°C for 5 min, followed by 30 cycles of 60°C for 30 sec, 72°C for 30 sec and 94°C for 30 sec. After amplification, a first cloning step was carried out using the TOPO TA Cloning kit (Invitrogen). The sequence of each VCR mutant sites was verified using an ABI BigDye Terminator v.3.1 sequencing kit and an ABI Prism 3100 Capillary Genetic Analyzer (Applied Biosystem) before being transferred, by *Eco*RI/*Bam*HI or *Mfe*I/*Bam*HI digestion (depending on the restriction sites present in each primer), into the pSW23T [36] and pSW23T_ISS_ [30] plasmids linearized by *Eco*RI/*Bam*HI digestion. Π1, a [Pir^+^] DH5α derivative that requires Thy to grow in MH medium, was used as a cloning strain.

### Suicide conjugation assays

#### The *in vivo* integration assay

This conjugation assay was based on that of Biskri et *al.* [32] and was previously implemented in Bouvier et *al.* [30]. In this assay, conjugation is used to deliver an *attC* recombination site in ss form into a recipient cell expressing the IntI1 integrase and carrying the *attI1* site. Briefly, the *attC* recombination sites provided by conjugation are carried on a suicide vector from the R6K based pSW family [36] that is known to use the Pir protein to initiate its own replication. This plasmid also contains an RP4 origin of transfer (*oriT*_RP4_). Two pSW derivatives, pSW23T and pSW23T_ISS_, allow the insertion of the tested *attC* sites in one orientation or the other compared to the *oriT*_RP4_, and transfer of respectively either the *attC*-bs or the *attC*-ts. The donor strain β2163 carries an RP4 integrated in its chromosome, requires DAP to grow in rich medium and sustains pSW replication through the expression of a chromosomally integrated *pir* gene. The recipient strain UB5201, which contains the pTRC99A::*intI1* [Ap^R^] and the pSU38-*attI1* [Km^R^], is devoid of a *pir* gene and therefore cannot sustain replication of the suicide vector. The only way for the pSW vector to be maintained in the recipient cell is to form a co-integrate by *attC*×*attI* recombination. The recombination activity is calculated as the ratio of transconjugants expressing the pSW marker [Cm^R^] to the total number of recipient clones [Ap^R^ Km^R^]. *attC*×*attI* co-integrate confirmation was confirmed and recombination point localization were performed as described below. Each recombination value represents a mean of 3 independent experiments. Standard deviations were within the same range that of those obtained with the same assay in our former studies [30],[31].

#### The *in vivo* deletion assay

This conjugation assay is based on the suicide conjugation assay previously described. In this assay, a synthetic cassette (*attC_aadA7_*-*lacI^q^*-VCR_2_) carried by a pSW suicide vector is transferred by conjugation to a recipient strain that expresses the IntI1 integrase. The synthetic cassette is inserted between a P*_tac_* promoter and a promoter-less pir116* gene [36] that encodes a functional Π protein ([P*_tac_*]-*attC_aadA7_*-*lacI^q^*-VCR_2_-pir116*). The pSW23T and pSW23T_ISS_ derivatives allow the transfer of either strand of the [P*_tac_*]-*attC_aadA7_*-*lacI^q^*-VCR_2_-pir116* extended cassette. The donor and recipient strains are those used for the previously described integration assay. In this assay, the recipient strain UB5201 only contains the pTRC99A::*intI1* [Ap^R^]. In the native [P*_tac_*]-*attC_aadA7_*-*lacI^q^*-VCR_2_-pir116* configuration, the Π protein cannot be expressed and the pSW vector cannot be maintained in the recipient strain. However, if the cassette is deleted through an *attC_aadA7_*×VCR_2_ recombination event catalyzed by IntI1, the pir116* gene becomes expressed from the P*_tac_* promoter, and the produced Π protein is able to sustain pSW replication in the recipient strain, which can be selected based on the pSW23 Cm^R^ marker (Figure 6). The deletion activity is calculated as the ratio of transconjugants expressing the pSW marker [Cm^R^] to the total number of recipient clones [Ap^R^]. *attC_aadA7_*×VCR_2_ recombination was confirmed by PCR using the Sw23begin and Sw23end primers (Table S1). The recombination point was precisely determined by sequencing using the same primers.

### Conjugation procedure

Conjugations were performed overnight on filter as previously described [30].

#### Measure of co-integrate formation and recombination point localization

*attC*×*attI* co-integrate formation was confirmed by PCR using the GoTaq Flexi DNA polymerase (Promega), according to the manufacturer′s instructions, and the MRV, MFD, Sw23begin and Sw23end primers (Table S1) on 8 recombinant clones randomly chosen in each mating experiments, giving a total of 24 clones sequenced for each tested strand of each mutant.

These primer sets allow discrimination between all possible recombination events. Indeed, when the VCR-bs is transferred, if it is recombined in its R′ box both the MRV/Sw23end or MFD/Sw23begin pairs of primers would amplify the co-integrate junctions. If, on the contrary, it is recombined in its R″ box then both the MRV/Sw23begin or MFD/Sw23end pairs of primers would amplify the co-integrate junctions (Figure 4). Conversely, when the *attC*-ts is transferred, if it is recombined in its R″ box both the MRV/Sw23end or MFD/Sw23begin pairs of primers would amplify the co-integrate junctions. If, on the contrary, it is recombined in its R′ box, either the MRV/Sw23begin or MFD/Sw23end pairs of primers would amplify the co-integrate junctions (Figure 4). In addition, the co-integrate junctions were sequenced using the Sw23begin and Sw23end primers.

## Supporting Information

Figure S1 Sequence alignment of the top strand of several natural *attC* sites. The R′, L′, L′, and R′ boxes are shown.
(0.06 MB PDF)

Table S1 Oligonucleotides used in this study
(0.22 MB DOC)
