# Supplementary material for: Structural Features of Single-Stranded Integron Cassette attC Sites and Their Role in Strand Selection
Source: PLoS Genet. 2009 Sep 4;5(9):e1000632. doi: 10.1371/journal.pgen.1000632 (PMC2727003; doi:10.1371/journal.pgen.1000632)
Supplement: Table S1 — Oligonucleotides used in this study (0.22 MB DOC) [file pgen.1000632.s002.doc]

**Table SI:** oligonucleotides used in this study

Sequences are given in 5’ 🡪 3’ direction. Restriction sites used for constructions are underlined.

1. DNA oligonucleotides used in the *in vivo* integration assay to generate the *attC* mutated sites.

| ***attC* sites** | | **Sequences** |
| --- | --- | --- |
| **mut1-VCR** | **rev** | GAATTCGGTTATAACAAACGCCTAAGGGGCTGTCAACGCGTGGCGTTTCCAGTCCCATTGAGCCGCGGTGGTTGCTGTTGTTGTGTT |
|  | **fwd** | GGATCCGGTTATAACGCCCGCCTCAAGAGGGACTGACAACGCACTACCACTAAACTCAAACACAACAACAGCAACCACCGCGGCTCAATGG |
| **mut2-VCR** | **rev** | GAATTCGGTTATAACAAACGCCTAAGAGGGACTGTCAACGCGTGGCGTTTCCAGTCCCATTGAGCCGCGGTGGTTGCTGTTGTTGTG |
|  | **fwd** | GGATCCGGTTATAACGCCCGCCTCAAGGGGCTGACAACGCACTACCACTAAACTCAAACACAACAACAGCAACCACCGCGGCTCAATGG |
| **mut3-VCR** | **rev** | GAATTCGGTTATAACAAACGCCTCAAGGGGACTGTCAACGCGTGGCGTTTCCAGTCCCATTGAGCCGCGGTGGTTGCTGTTGT |
|  | **fwd** | GGATCCGGTTATAACGCCCGCCTAAGAGGGCTGACAACGCACTACCACTAAACTCAAACACAACAACAGCAACCACCGCGGC |
| **mut4-VCR** | **rev** | GAATTCGGTTATAACAAACGCCTCAAGAGGGCTGTCAACGCGTGGCGTTTCCAGTCCCATTGAGCCGCGGTGGTTGCTGTTGT |
|  | **fwd** | GGATCCGGTTATAACGCCCGCCTAAGGGGACTGACAACGCACTACCACTAAACTCAAACACAACAACAGCAACCACCGCGGC |
| **mut5-VCR** | **rev** | GAATTCGGTTATAACAAACGCCTAAGGGGACTGTCAACGCGTGGCGTTTCCAGTCCCATTGAGCCGCGGTGGTTGCTGTTGTTGTG |
|  | **fwd** | GGATCCGGTTATAACGCCCGCCTCAAGAGGGCTGACAACGCACTACCACTAAACTCAAACACAACAACAGCAACCACCGCGGCTCAATGGG |
| **mut6-VCR** | **rev** | GAATTCGGTTATAACAAACGCCTAAGAGGGCTGTCAACGCGTGGCGTTTCCAGTCCCATTGAGCCGCGGTGGTTGCTGTTGT |
|  | **fwd** | GGATCCGGTTATAACGCCCGCCTCAAGGGGACTGACAACGCACTACCACTAAACTCAAACACAACAACAGCAACCACCGCGGCTC |
| **mut7-VCR** | **rev** | GAATTCGGTTATAACAAACGCCTCAAGGGGCTGTCAACGCGTGGCGTTTCCAGTCCCATTGAGCCGCGGTGGTTGCTGTTGT |
|  | **fwd** | GGATCCGGTTATAACGCCCGCCTAAGAGGGACTGACAACGCACTACCACTAAACTCAAACACAACAACAGCAACCACCGCGGC |
| **mut8-VCR** | **rev** | GAATTCGGTTATAACAAACGCCTAAGGGGCTGTCAACGCGTGGCGTTTCCAGTCCCATTGAGCCGCGGTGGTTGCTGTTGTTGTGTT |
|  | **fwd** | GGATCCGGTTATAACGCCCGCCTCAAGGGGCTGACAACGCACTACCACTAAACTCAAACACAACAACAGCAACCACCGCGGCTCAATGG |
| **mut9-VCR** | **rev** | GAATTCGGTTATAACAAACGCCTCAAGGGGCTGTCAACGCGTGGCGTTTCCAGTCCCATTGAGCCGCGGTGGTTGCTGTTGTTGT |
|  | **fwd** | GGATCCGGTTATAACGCCCGCCTAAGGGGCTGACAACGCACTACCACTAAACTCAAACACAACAACAGCAACCACCGCGGCTCAATG |
| **mut10-VCR** | **rev** | GAATTCGGTTATAACAAACGCCTTAAGAGGGACTGTCAACGCGTGGCGTTTCCAGTCCCATTGAGCCGCGGTGGTTGCTGTTGTTGT |
|  | **fwd** | GGATCCGGTTATAACGCCCGCCTAAGGGGCTGACAACGCACTACCACTAAACTCAAACACAACAACAGCAACCACCGCGGCTCAATG |
| **mut11-VCR** | **rev** | GAATTCGGTTATAACAAACGCCTAAAGAGGGACTGTCAACGCGTGGCGTTTCCAGTCCCATTGAGCCGCGGTGGTTGCTGTTGTTGT |
|  | **fwd** | GGATCCGGTTATAACGCCCGCCTAAGGGGCTGACAACGCACTACCACTAAACTCAAACACAACAACAGCAACCACCGCGGCTCAATG |
| **mut12-VCR** | **rev** | GAATTCGGTTATAACAAACGCCTGAAGAGGGACTGTCAACGCGTGGCGTTTCCAGTCCCATTGAGCCGCGGTGGTTGCTGTTGTTGT |
|  | **fwd** | GGATCCGGTTATAACGCCCGCCTAAGGGGCTGACAACGCACTACCACTAAACTCAAACACAACAACAGCAACCACCGCGGCTCAATG |
| **mut13-VCR** | **rev** | GAATTCGGTTATAACAAACGCCTCAAGAGGGACTGTCAACGCGTGGCGTTTCCAGTCCCATTGAGCCGCGGTGGTTGCTGTTGTTGTGT |
|  | **fwd** | GGATCCGGTTATAACAAACGCCTAAGGGGCTGACAACGCACTACCACTAAACTCAAACACAACAACAGCAACCACCGCGGCTCAAT |
| **mut14-VCR** | **rev** | GAATTCGGTTATAACAAACGCCTCAAGAGGGACTGTCAACGCGTGGCGTTTCCAGTCCCATTGAGCCGCGGTGGTTGCTGTTGTTGTGTT |
|  | **fwd** | GGATCCGGTTATAACCCCCGCCTAAGGGGCTGACAACGCACTACCACTAAACTCAAACACAACAACAGCAACCACCGCGGCTCAATGG |
| **mut15-VCR** | **rev** | GAATTCGGTTATAACAAACGCCTCAAGAGGGACTGTCAACGCGTGGCGTTTCCAGTCCCATTGAGCCGCGGTGGTTGCTGTTGTTGTGTT |
|  | **fwd** | GGATCCGGTTATAACACCCGCCTAAGGGGCTGACAACGCACTACCACTAAACTCAAACACAACAACAGCAACCACCGCGGCTCAATGG |
| **mut16-VCR** | **rev** | GAATTCGGTTATAACAAACGCCTCAAGAGGGACTGTCAACGCGTGGCGTTTCCAGTCCCATTGAGCCGCGGTGGTTGCTGTTGTTGTGTT |
|  | **fwd** | GGATCCGGTTATAACTCCCGCCTAAGGGGCTGACAACGCACTACCACTAAACTCAAACACAACAACAGCAACCACCGCGGCTCAATGG |
| **mut17-VCR** | **rev** | GAATTCGGTTATAACGCCCGCCTAAGGGGCTGTCAACGCGTGGCGTTTCCAGTCCCATTGAGCCGCGGTGGTTGCTGTTGTTG |
|  | **fwd** | GGATCCGGTTATAACAAACGCCTCAAGAGGGACTGACAACGCACTACCACTAAACTCAAACACAACAACAGCAACCACCGCGG |
| **mut18-VCR** | **rev** | GAATTCGGTTATAACGCCCGCCTAAGGGGCTGTCAACGCGTGGCGTTTCCAGTCCCATTGAGCCGCGGTGGTTGCTGTTGTTG |
|  | **fwd** | GGATCCGGTTATAACAAACGCCTCAAGAGGGACTGACAACGCACTACCACTAAACTCAAACACAACAACAGCAACCACCGCGG |
| **mut19-VCR** | **rev** | GAATTCATCCGGTTATAACAAACGCCTAAGGGGCTGTCAACGCGTGGCGTTTCCAGTCCCATTGAGCCGCGGTGGTTGCTGTTGT |
|  | **fwd** | GGATCCATCCGGTTATAACGCCCGCCTAAGGGGCTGACAACGCACTACCACTAAACTCAAACACAACAACAGCAACCACCGCGGCTC |
| **mut20-VCR** | **rev** | GAATTCGGTTATAACGCCCGCCTAAGGGGCTGTCAACGCGTGGCGTTTCCAGTCCCATTGAGCCGCGGTGGTTGCTGTTGTTGTGTTTGA |
|  | **fwd** | GGATCCAAGGTTATAACAAACGCCTAAGGGGCTGACAACGCACTACCACTAAACTCAAACACAACAACAGCAACCACCGCGGCTCAATGG |
| **mut21-VCR** | **rev** | GAATTCGGTTATAACAAACGCCTAAGGGGCTGTCAACGCGTGGCGTTTCCAGTCCCATTGAGCCGCGGTGGTTGCTGTTGTTGTGTTTGA |
|  | **fwd** | AAGCTTGGATCCATCCGGTTATAACGCCCGCCTAAGGGGCTGACAACGCACTACCACTAAACTCAAACACAACAACAGCAACCACCGCGGCTC |
| **mut22-VCR** | **rev** | GAATTCAGATCTGTTATAACAAACGCCTCAAGAGGGACTGTCAACGCTTCGCGTTGTCAGCCCCTTAGGCGG |
|  | **fwd** | GGATCCGGTTATAACGCCCGCCTAAGGGGCTGACAAC |
| **mut23-VCR** | **rev** | GAATTCGGTTATAACAAACGCCTCAAGAGGGACTGTCAACGCGAAGCGTTGTCAGCCCCTTAGGCGG |
|  | **fwd** | GGATCCGGTTATAACGCCCGCCTAAGGGGCTGACAAC |
| **wt-ereA2** | **rev** | GAATTCGCATAACCTGCCAATCCACCGGACGGTTTTCAACCGCCGG |
|  | **fwd** | GGATCCGCATAACGCGCTGATCACCGGCGGTTGAAAACCGTCC |
| **mut24-ereA2** | **rev** | GAATTCCGCATAACCTGCCAATCACCGGCGGTTTTCAACCGTCCGG |
|  | **fwd** | GGATCCGCATAACGCGCTGATCCACCGGACGGTTGAAAACCGCC |
| **wt-oxa2** | **rev** | GAATTCGCCCAACCCGGCAGTCAACTCGGACGCTGCGCGATAAAACC |
|  | **fwd** | GGATCCGCCCAACGTTGAAGTAACCGGCGCTGCGCGGTTTTATCGCGCAGCGTCCG |
| **mut25-oxa2** | **rev** | GAATTCCGCCCAACCCGGCAGTAACCGGCGCTGCGCGATAAAACCGCGCAGC |
|  | **fwd** | GGATCCGCCCAACGTTGAAGTCAACTCGGACGCTGCGCGGTTTTATCGCGCA |

B) DNA oligonucleotides used in the *in vivo* deletion assay

| **Name** | **Sequences** |
| --- | --- |
| **pirA1** | CCGGAATTCGCATGCTTAACAGGAGCTAAGGAAGCTAAAATGAGACTCAAGGTCATGATGGACGT |
| **pirA2** | ATGAATACTGGCTACTTAAGCGTAGGCTTGCCGTTAACAATTGCGTTGTG |
| **pirB1** | CACAACGCAATTGTTAACGGCAAGCCTACGCTTAAGTAGCCAGTATTCAT |
| **pirB2** | CTTCAGATAAATTCATAAAAAATGCTTCATCATCTTTATCGCCAGAAAATTC |
| **pirC1** | AAGCATTTTTTATGAATTTATCTGAAGCTGATGCAGCTTTTCTCAAGGTATTTGATGAAACCGT |
| **pirC2** | GCGGGATCCAAGCTTACCCCTTAGCTTTTTTGGGAGGTACGGTTTCATCAAATACCTTGAGAA |
| **1187-ΔCat-1** | GATATCGGTACCAAAAAAAAGCCCGCTCATTAGGCGGGCTCGAATAAATACCTTTTTTTAAGGCAGTGGATCCCATCTA |
| **1187-ΔCat-2** | GATATCTCTAGACCATCTAACAAACGCCTCAAGAGGGACTG |
| **lacI^q^-1** | GCTCTAGAGGCGAAGCGGCATGCATTTAC |
| **lacI^q^-2** | CGGGGTACCGCTGTCAAACCAGATCAATTCGC |
| **pTAC(pTX)-1** | CGCGGATCCGAGCTCGCACGACAGGTTTCCCGACTG |
| **pTAC(pTX)-2** | CCGCAATTGAAGCTTTTGTGCAATACCGGAATTCAGCGGCCGCAAATTCC |
| **VCR(ΔEHB)** | CGCCACGCGTTGACAGCCCCTTAGGCGTTTGTTAGATGGTCTAGAGG |
| **SDM-VCR-ΔEHB** | CCTAAGGGGCTGTCAACGCGTGGCGTTTCCAGTCCCATTG |
| **Ad7-ΔEHB-1** | GAATTCGGTTATAACAATTCATTAAGCCGCGCCGCTTCGCGGCGCGGCTTAATTCAAGC |
| **Ad7-ΔEHB-2** | GGATCCCATCTAACGCTTGAATTAAGCCGCGCCGC |
| **VCR(G_16_🡪C)** | CACGCGTTGACAGTCCCTCTTCAGGCGTTTGTTAGATGGTCTAGA |
| **VCR(G_16_🡪T)** | CACGCGTTGACAGTCCCTCTTTAGGCGTTTGTTAGATGGTCTAGA |
| **VCR(G_16_🡪A)** | CACGCGTTGACAGTCCCTCTTAAGGCGTTTGTTAGATGGTCTAGA |
| **SDM-VCR** | AAGAGGGACTGTCAACGCGTGGCGTTTCCAGTCCCATTG |

C) DNA oligonucleotides used to confirm on one hand the *attC* x *attI* insertion and on the other hand the *attC* x *attC* deletion events.

| **Name** | **Sequences** |
| --- | --- |
| **MRV** | AGCGGATAACAATTTCACACAGGA |
| **MFD** | CGCCAGGGTTTTCCCAGTCAC |
| **Sw23begin** | CCGTCACAGGTATTTATTCGGCG |
| **Sw23end** | CCTCACTAAAGGGAACAAAAGCTG |
