# Supplementary material for: Structural Features of Single-Stranded Integron Cassette attC Sites and Their Role in Strand Selection
Source: PLoS Genet. 2009 Sep 4;5(9):e1000632. doi: 10.1371/journal.pgen.1000632 (PMC2727003; doi:10.1371/journal.pgen.1000632)
Supplement: Figure S1 — Sequence alignment of the top strand of several natural attC sites. The R′, L′, L′, and R′ boxes are shown. (0.06 MB PDF) [file pgen.1000632.s001.pdf]

```
ereA2       TAACCTGCCAATCCACCG--GACGGTT--------------------------------------TTC------------------------------------------------AACCGCCGGTGATCAGCGCGTTA
oxa2        TAACCCGGC-AGTCAACTCGGACGCTGCGCG--------------------------------ATAAAAC-----------------------------------------CGCGCAGCGCCGGTTACT-TCAACGTTA
aadA5       TAACTCGGC-GTTCAAG-CGGACGGGC--------------------------------------TGC-----------------------------------------------GCCCGCCGCTCAAC-TATGCGTTA
oxa9        TAACCCTGC-GCTCGAG-CGGACCTCGCGC---------------------------------ATAAAGCC-----------------------------------------GCGCGAGTCCGCTCACCTTGAACGTTA
dfrB1       TAACTTGTC-GCTCCAG-CGGACGGC---------------------------------------TTC-----------------------------------------------GCCGCCCGCTGAGCTAATTCGTTA
dfrB3       TAACTGGTC-GCTCCAG-CGGACGGC---------------------------------------TTC-----------------------------------------------GCCGCCCGCTGAGCTAGAGCGTTA
aacA-IIb    TAACTTTTCGCTCCAA--CGGACGCTTGACCCGTCGGCCCGGCCGC------------------TGCC----------------------------GCAGCCGGACCGCCGCGTCAACCGCCGCTGACCTCCGGAGTTA
dfrA15      TAACAAGTGGCAGCAA--CTGACCGCCAAAAGTGTCACTTGTTTTGCC---------------AAAAAGCC-----------------------GGCAAAACAAGCGCCAATTTTGTCGGCAGCTGTG-CCAGGCGTTA
dfrA16      TAACAAGCGCCTGCAA-TCTGACCTCCGGTTACTGTCACCTTTTTTTGCGG-------------TGGAG------------------------CTGCAAAAAAGGCGCCATTAACCTCCGGCAGTTGAGGCGGGCGTTA
dfrA1       TAACAAGTGGCAGCAA--CGGATTCGCAAA-CCTGTCACGCCTTTT------------------GTGCC----------------------------AAAAGCCGCGCCAGGTTTGCGATCCGCTGTG-CCAGGCGTTA
blaP1       TAACAAGGC-CATCAAGTTG-ACGGCTTTT-CC-GTCGCTTGTTTTGTGGT------------TTAACGCT------------------ACGCTACCACAAAACAATCAACTCCAAAGCCGCAACTTATGGCGGCGTTA
blaP2       TAACAAGGC-CATCAAGTTG-ACGGCTTTT-CC-GTCGCTTGTTTTGTGG----------CTTAACGCTACGCTA--------------------CCACAAAACAATCAACTACAAAGCCGCAACTTATGGCGGCGTTA
aadA6       TAACAATTC-ATTCAAGCCG-ACGCCGC-------------------------------------TTC----------------------------------------------GCGGCGCGGCTTAATTCAGGCGTTA
catB3       TAACAATTC-AATCAAGCCG-ATGCCGC-------------------------------------TTC----------------------------------------------GCGGCACGGCTTATTTCAGGCGTTA
catB5       TAACAATTC-ATTCAAGCCG-ACGCCGC-------------------------------------TTC----------------------------------------------GCGGCACGGCTTAATTCTGGCGTTA
qacF        TAACAATTC-ATTCAAGCCG-ACGCCGC-------------------------------------TTC----------------------------------------------GCGGCGCGGCTTAATTCAGGCGTTA
aadA7       TAACAATTC-ATTCAAGCCG-ACGCCGC-------------------------------------TTC----------------------------------------------GCGGCGCGGCTTAATTCAAGCGTTA
aacA-IIa    TAACAACTC-ATTCAAGCCG-ACGCCGC-------------------------------------TTC----------------------------------------------GCGGCGCGGCTTAATTCAGGCGTTA
sat         TAACAATTC-ATTCAAGCCG-ACACCGC-------------------------------------TTC----------------------------------------------GCGGCGCGGCTTAATTCAGGAGTTA
aadA1a      TAACAATTC-GTTCAAGCCG-ACGCCGC-------------------------------------TTC----------------------------------------------GCGGCGCGGCTTAACTCAAGCGTTA
aadB        TAACAATTC-GTCCAAGCCG-ACGCCGC-------------------------------------TTC----------------------------------------------GCGGCGCGGCTTAACTCAGGTGTTA
aadA1b      TAACAATTC-GTTCAAGCCG-ACGCCGC-------------------------------------TTC----------------------------------------------GCGGCGCGGCTTACCTTGGCCGTTA
aadA3       TAACAATTC-GTTCAAGCCG-ACGCCGC-------------------------------------TTC----------------------------------------------GCGGCGCGGCTTAACTCAAGCGTTA
arr2        TAACAAAGC-CATCAAACCGGACGCCAGAGATTCCGCGCCTGTTGCGCATGGC------------TTC---------------------GCATATGCGCAATAGGCG-CGCCACCCTGTCGCCGTTTATGGCGGCGTTA
aacA-Iq     TAACAACCG-CTTCAAC-CTGAC-TCAGGGCGCTGTCACGATTTCTGCAGG--------------TAAT-------------------TCTGGACGCAGAAATCGCGCCAACGCCCTTCGCAGGTTAAGCGAAGTGTTA
aacA-Ip     TAACAATCG-CTTCAAC-CTGAC-TCAGGGCGCCGTCACGATTTCTGCTAGTT----------ATCCTGG----------------------GACGCAGAAATCGCGCCAACGCCCTTCGCAGGTTAAGCGAAGTGTTA
aacA4b      TAACCCTTC-CATCGAGGGGGACGTCCAAGG--------------------------------GCTGGCG-----------------------------------------CCTTGGCCG-CCCTCATGTCAAACGTTA
aacA4a      TAACCCTTC-CATCGAGGGGGACGTCCAAGGGC--------------------------------TGGC----------------------------------------GCCCTTGGCCGCCCCTCATGTCAAACGTTA
orfB        TAACCCTTC-CATCGAG-GGGATGCCCAAAAGGC-------------------------------TGC----------------------------------------GCCTTCTGTGCACCCCTCATGTCAAACGTTA
dfrA12      TAACCATTC-CGTCAAC-GGGACGCCAAAATGC--------------------------------TGC--------------------------------GCATTTTGGTTCCCTCCGCTGCGCTCCGGC-TCTCGTTA
aacC1       TAACAATTC-GTTCAAGCCG-AGATCGGCTTCCCGGCCGCGGAGTTGTTCGG-------------TAAA---------------------TTGTCACAACGCCGCGGCCGCAAAGCGCTCCGGCTTAACTCAGGCGTTA
aadA2       TAACAATTC-GTTCAAGCCG-ACCGCGC-------------------------------------TAC----------------------------------------------GCGCGGCGGCTTAACTCCGGCGTTA
aacA7       TAACAATTC-GTTCAAGCCGAACTTGCTTCGTTACACCAAAGCCATGGCAG------------AAAGAGC--------------------TTGCCATGGCTTTGGCTCCACTACGCAAGTCGGCTTAACTCAGGCGTTA
orfD        TAACATTTC-GGTCAAGCCG-ACCCGCA-------------------------------------TTC---------------------------------------------TGCG-GTCGGCTTACCTCGCCCGTTA
blaVIM2     TAACATGAAGTTGCA-GCCG-ACCATCACTCCGC-------------------------------TGC----------------------------------------GCTCCGTTCTGGCGGCTGAACTTCGGCGTTA
dfrB2       TAACAATTCGCTCCA-G-CGGACGGC---------------------------------------TTC-----------------------------------------------GCCGCCCGCTGAGCTTTATCGTTA
oxa7        TAACAATTCGCTGCAGGCGCGACGGCCC-------------------------------------TGAC-------------------------------------------GGGCCGCGGCCTGAGCTCAAACGGTTA
cmlA        TAACAAATCGCTGCA-GCCG-ACCCAAAACCGC--------------------------------TAC-----------------------------------------GCGGTTTCGGTCGGCTGAGCTCAGGCGTTA
cmlA2       TAACAAATCGCTGCA-GCCG-ACCCAAAACCGC--------------------------------TAC-----------------------------------------GCGGTTTCGGTCGGCTGAGCTCAAGCGTTA
cmlA4       TAACAAATCGCTGCA-GCCG-ACCCAAAACCGC--------------------------------TAC-----------------------------------------GCGGTTTCGGTCGGCTGAGCTCAGGCGTTA
dfrA14      TAACAAAGCTATGCAAT-CG-ACGGCAAAAAGCTTCGTTCGC-----------------------TTC-------------------------------GCGCACTACGCT-TTTT-CCGC-GATTGATAGCGACGTTA
dfrA5       TAACAAAGCTATGCAATTG--ACGGTAAAAAGCTTCGTTCGC-----------------------TTC-------------------------------GCTTGCTACGCT-TCTTACCGC-AATTGATAACGGCGTTA
blaP3       TAACAAAGCATTT-AAGAGGGACAGCCAACGCGTGGCACTTTTATTAT-------------GCGTTGGTTTTT----GTGGTTACGGTGTTATGCGGTAAGTTGGTAGTAGCGTTGGCTGCCCCTTAAT-GCGGCGTTA
VCR2/1      TAACAAAC-GCCTCAAGAGGGACTGTCAACGCGTGGCGTTTCCAGTCCCATTGAGCCGCGGTGGTTGCTGTTG-----------TTGTGTT-TGAGTTTAG-TGGTAGT-GCGTTGTCAGCCCCTTAGG-CGGGCGTTA
blaVIM1     TAACAAATCGTTGGAGC-GGGACTTTTGCTACGCAGGC---------------------------TGC------------------------------------GCCTACTCCGCAAAAGCCCCTCAACTCAGGCGTTA

             R”           L”                                                                                                               L’           R’
```

- **Figure S1** -

Sequence alignment of the top strand of various attC sites. The inverted repeats L‘, L‘‘, R’ and R‘’ are boxed
